# Quick Sequential Organ Failure Assessment to identify clinical deterioration in adults with COVID-19: a retrospective cohort*

**DOI:** 10.1590/1518-8345.7239.4530

**Published:** 2025-05-19

**Authors:** Luiz Felipe Sales Mauricio, Cassia Regina Vancini Campanharo, Luiz Humberto Vieri Piacezzi, Maria Carolina Barbosa Teixeira Lopes, Ruth Ester Assayag Batista

**Affiliations:** 1 Universidade Federal de São Paulo, Escola Paulista de Enfermagem, São Paulo, SP, Brazil

**Keywords:** COVID-19, Vital Signs, Early Warning Score, Clinical Deterioration, Nursing Care, Hospital Emergency Service

## Abstract

to evaluate the performance of qSOFA in identifying deterioration in patients with COVID-19.

retrospective cohort study conducted between February and August 2020 in the Emergency Department of a private hospital, involving 813 adults. The variables studied included sociodemographic data, clinical characteristics, deterioration, qSOFA on admission and before the event, and outcomes. The performance of qSOFA at both moments was analyzed using the area under the ROC curve.

the average age was 69 years. There was a predominance of men (61.5%), white (97.2%), catholic (73.7%), married (89.6%) and employed (66%). Comorbidities were present in 69.7%, and 58.8% were classified as “urgent” upon admission. The most frequent deterioration was respiratory failure (16.7%), and the outcome was discharge (68%). Patients with positive qSOFA on admission had a higher percentage of respiratory failure, cardiopulmonary arrest, and “very urgent” risk classification, and those with negative qSOFA showed a higher percentage of discharge (p< 0.0001). Upon admission, qSOFA showed 66% sensitivity and 55% specificity, and prior to the event it showed 48% sensitivity and 88% specificity for identifying clinical deterioration. Patients with positive qSOFA on admission were 350 times more likely to experience deterioration.

qSOFA showed low sensitivity for identifying deterioration at both moments and high specificity before the event.

## Highlights

**(1)** Patients with COVID-19 and positive qSOFA had a higher risk of death. **(2)** Positive qSOFA detected early clinical deterioration in COVID-19 patients. **(3)** Patients with COVID-19 and positive qSOFA used more diagnostic resources.

## Introduction

The SARS-COV-2 virus, responsible for the disease COVID-19, was identified in the People’s Republic of China in late 2019^(1)^, spreading rapidly throughout the world^(2-3)^, and in March 2020 the World Health Organization (WHO) declared the pandemic^(1)^. As of December 2022, 662,413,265 cases and 6,687,694 deaths caused by COVID-19 had been confirmed worldwide^(4)^. In Brazil, in the same period, there were 36,202,186 confirmed cases and 693,017 deaths^(4)^.

COVID-19 has distinct clinical profiles, from asymptomatic infection to critical clinical conditions. The most frequent signs and symptoms are fever, fatigue and dry cough; however, some cases may progress to respiratory failure, requiring ventilatory support, septic shock/sepsis, renal failure, thromboembolism and cardiopulmonary arrest (CPA)^(2,4)^, which makes early identification of signs of clinical deterioration mandatory.

In this context, the assessment of the patient’s clinical condition is extremely important, and must begin with the risk classification to manage the correct flow of care and immediate care in life-threatening conditions^(2)^.

However, early identification scores for clinical deterioration, which is defined as the worsening of an individual’s clinical condition, manifested by changes in vital signs, mental status, or other clinical parameters, which indicate a potential threat to life or the need for urgent medical intervention^(5)^, have been used with the aim of complementing clinical assessment in the emergency department. Identifying an accurate score to track clinical deterioration can improve the quality and safety of care. The quick Sequential Organ Failure Assessment (qSOFA) has been a recommended score to identify patients with infection at high risk of poor outcomes in contexts outside the intensive care unit (ICU)^(6)^. Summarized from the Sequential Organ Failure Assessment (SOFA), a well-established score for risk stratification in patients with sepsis, qSOFA is a simple alert tool, applicable in any context, with the aim of identifying clinical signs of deterioration related to sepsis^(7)^. By analyzing only 3 clinical signs – respiratory rate, systolic blood pressure and mental status –, qSOFA performed well in a sepsis scenario^(8)^. In a meta-analysis that included six studies with 17,868 patients, qSOFA was found to be a good predictor of pneumonia mortality^(8)^. In another review, qSOFA performed well in predicting mortality in patients with or without infectious signs^(9)^.

As pneumonia is the main complication in COVID-19 and can quickly progress to respiratory failure, qSOFA can be useful for identifying clinical worsening in a simple and early manner. A prompt and systematic assessment will detect signs of clinical instability, enabling rapid intervention with a consequent increase in patient survival^(6)^. Although no studies were found recommending a specific early warning system (EWS) for patients with COVID-19, it is reasonable to assume that its use can improve the quality of vital signs documentation, assisting in the systematic and recurring assessments by nurses.

Not only during the COVID-19 pandemic, for more than a decade, emergency services have had overcrowding as one of the main challenges for maintaining the quality and safety of care. In the face of the increased demand for patients with severe clinical conditions at risk of death, there is an emerging need to identify early predictors of clinical deterioration in order to reduce mortality through the timely implementation of appropriate therapy. However, nursing research on this topic is still in its early stages.

Finally, evaluating which score performs better in a given scenario seems pertinent. Therefore, this case study aims to assess the performance of qSOFA in predicting clinical deterioration in patients hospitalized with COVID-19.

## Method

The methodological report of this study was carried out in accordance with the guidelines of Strengthening the Reporting of Observational Studies in Epidemiology (STROBE)^(10)^.

### Study design and period

Retrospective cohort study conducted from February 1^st^ to August 3^rd^, 2020, during the first peak of the pandemic in Brazil, in the Emergency Department (ED) of a private hospital in the Southeast region of Brazil.

### Population

All patients admitted to the ED during the study period were included, with the following criteria: over 18 years old, diagnosed with COVID-19 and with completeness of the variables of interest. Due to their distinct clinical characteristics, patients undergoing palliative care were excluded, as data collection involved variables that contained medical or nursing interventions with the aim of curing the disease or prolonging the patient’s life; and pregnant women, for whom, due to undergoing physiological changes specific to pregnancy, qSOFA does not have robust validation.

### Data collection

The hospital’s Epidemiology Department provided an MS Excel^®^ spreadsheet with a list of all medical record numbers of patients admitted to the ED with a diagnosis of COVID-19 during the study period. With this list in hand, as the country was still in the midst of the pandemic, the Free and Informed Consent Form (FICF) was sent by email. Participants were included in the study after expressing their willingness to participate by signing the FICF electronically. In cases of patients who died or who did not return after three attempts to contact them and who were not being followed up at the institution, an exemption was obtained for the application of the FICF. After authorization from research participants, electronic medical records were accessed from September 2021 to February 2022, and data were collected by the principal researcher and stored on the Research Electronic Data Capture (REDCap^®^) plataform^(11)^, as recommended by the institution. The security, secrecy and confidentiality of data were preserved by identifying patients using codes in the database.

### Study variables

All patient data were extracted exclusively from the electronic medical records of research participants. The sociodemographic variables collected were: age, gender, ethnicity, religion and marital status. The clinical variables were: time from onset of symptoms until seeking medical care, presence of comorbidities, risk classification category, level of consciousness, signs and symptoms.

The primary outcome of this study was clinical deterioration, defined as the documented need for interventions to restore homeostasis, including: introduction or increase of oxygen support, escalation of ventilatory modality, initiation of a new vasoactive drug, or performance of cardiopulmonary resuscitation. Furthermore, it was recorded whether or not the patient presented any of the clinical deteriorations in the first 24 hours.

The secondary outcomes were: discharge, hospitalization in a room, hospitalization in a semi-intensive care unit, hospitalization in an intensive care unit (ICU) and death in the first 24 hours. And the final outcomes were: discharge and death.

The institution where the study was conducted uses the Emergency Severity Index (ESI) as a risk classification system in the ED. In this protocol, the risk classification categories are: 1, 2, 3, 4 and 5. Levels 1 and 2 correspond to patients who are in emergency situations, that is, at risk of death; at levels 3, 4 and 5, patients are classified by the need for resources, such as complementary diagnostic tests, therapeutic procedures to be used and frequency of assessment of vital signs. Patients in category 3 are classified as urgent, in 4 as not very urgent, and in 5 as non-urgent^(12)^.

qSOFA was calculated at two times: on admission to the ED, during the risk classification carried out by the nurse, who evaluates and enters the values in the electronic medical record, with the calculation being performed automatically by the system; and in the case of clinical deterioration (CD) in the first 24 hours after admission to the ED, with the calculation being performed based on the last parameters recorded in the medical record by the professional responsible for patient care. qSOFA is composed of the following variables: respiratory rate, level of consciousness and systolic blood pressure^(6)^. One point should be awarded if the respiratory rate is greater than 22 breaths per minute, if there is an alteration in the level of consciousness (Glasgow Coma Scale score less than 15), or if the systolic blood pressure is less than 100 mmHg^(6)^. qSOFA is defined as positive when two or more variables are scored, and negative when one or none are scored^(6)^. The highest score (3 points) is associated with a poor prognosis^(13)^. When the score is ≥ 2, studies suggest it as an indicator of potential sepsis in patients with suspected infection outside the ICU^(13)^.

### Data processing and analysis

The research data were stored on the REDCap®^(11)^ collection platform and the Statistical Package for the Social Sciences (SPSS), version 23, was used to process the analysis. The descriptive analysis was performed by calculating the mean, standard deviation, median, minimum and maximum. For categorical variables, frequency and percentage were calculated. The association of qSOFA (positive or negative) with the occurrence of clinical deterioration, comorbidity, by risk classification category, outcome within 24 hours of admission and final outcome was verified using the chi-square test, and, when necessary, Fisher’s exact test was used. The Mann-Whitney test or Kruskal-Wallis test was used for 3 or more categories. The sensitivity and specificity of qSOFA for the occurrence of clinical deterioration were calculated. For all analyses, a p-value < 0.05 was considered for statistical significance. Simple logistic regression was performed to verify the relationship between independent variables and clinical deterioration (dependent variable). Multiple logistic regression was performed to verify the factor that best explained “clinical deterioration”. The selection method used was Forward. The performance of qSOFA for identifying clinical deterioration on admission and before the event was analyzed using the area under the ROC curve.

### Ethical aspects

This research complies with Resolution 466/2012 of the National Health Council and was approved by the institution’s Research Ethics Committee, opinion 4,919,847, Certificate of Presentation for Ethical Consideration (CAAE, in Portuguese): 32684620.3.3001.0071, and by the Research Project Management System of the co-participating institution, under opinion nº 4316-20.

## Results

In this study, there was no loss to follow-up. A total of 813 patients were included, with a mean age of 69 years (± 17.8), and with a prevalence of males (61.5%, n= 500), whites (97.2%, n=769), catholics (73.7%, n=583), married (89.6%, n= 578) and employed (66%, n= 456). The majority of the study population (75.3%, n= 612) sought medical care between the first and sixth day of symptom onset, 21.6% (n= 176) between the seventh and tenth, and 3.1% (n= 25) with more than 10 days of symptoms.

Regarding risk classification, of the total of 813 (n= 100%) patients, 58.8% (n= 478) were classified as ESI 3, 21.9% (n= 178) ESI 2, 18% (n= 146) ESI 4 and 1.4% (n= 11) ESI 1.

As for the occurrence of clinical deterioration, 16.7% (n= 136) of individuals presented acute respiratory failure in the first 24 hours of admission to the ED, 0.7% (n= 6) shock, 2.7% (n= 22) CPA and 2.8% (n= 23) altered level of consciousness. There were 187 clinical deteriorations in the first 24 hours of admission to the ED, according to the data in Table 1.

**Table 1 -:** Clinical deterioration of patients with COVID-19 in the first 24 hours after admission to the Emergency Department (n* = 813). São Paulo, SP, Brazil, 2022

**Clinical deterioration**	**24 hours after admission**
	n* (% ^†^ **)**
**Acute respiratory failure**	
No	677 (83.3)
Yes	136 (16.7)
**Shock**	
No	807 (99.3)
Yes	6 (0.7)
**Cardiopulmonary arrest**	
No	791 (97.3)
Yes	22 (2.7)
**Altered level of consciousness**	
No	790 (97.2)
Yes	23 (2.8)

*n = Absolute number; †% = Percentage

Of the studied population (n= 813), 69.7% (n= 567) had at least one comorbidity, with 41% (n= 332) being cardiovascular, 34.4% (n= 280) respiratory, 20.4% (n= 166) endocrine, and 10.8% (n= 88) neurological.

Individuals with comorbidities had a higher percentage of respiratory failure, CPA and altered level of consciousness than those without comorbidities (p< 0.0001). Of the individuals who had some type of comorbidity, 106 (n= 18.7%) presented respiratory failure, 6 (n= 1.1%) shock, 22 (n= 3.9%) cardiopulmonary arrest and 20 (n=3.5%) altered level of consciousness. It was observed that patients with comorbidities had a higher percentage of clinical deterioration compared to those without (p< 0.0001), as well as higher rates of acute respiratory failure (p= 0.0019), shock (p= 0.0036), CPA (p= 0.0083), and altered level of consciousness (p= 0.0007) when compared to those without comorbidities (p< 0.0001). Patients with comorbidities had a higher percentage of hospitalization in semi-intensive care (p< 0.0001) and ICU (p< 0.0001), and a higher frequency of death (p< 0.0001). Individuals without comorbidities had more hospital discharges when compared to those with comorbidities (p< 0.0001) (Table 2).

**Table 2 -:** Association of the presence of comorbidities with clinical deteriorations and outcomes in the studied population (n* = 813). São Paulo, SP, Brazil, 2022

**Variables**	**Presence of comorbidities**		**p** ^†^ **-value**
	No n* (% ^‡^ **)**	Yes n* (% ^‡^ **)**	
**Clinical deterioration on admission**			
No	213 (86.6)	413 (72.8)	<0.0001
Yes	33 (13.4)	154 (27.2)	
**Respiratory failure**			
No	214 (87)	440 (77.6)	0.0019
Yes	32 (13)	127 (22.4)	
**Shock**			
No	244 (99.2)	539 (95.1)	0.0036
Yes	2 (0.8)	28 (4.9)	
**Cardiopulmonary arrest**			
No	235 (95.5)	510 (89.9)	0.0083
Yes	11 (4.5)	57 (10.1)	
**Altered level of consciousness**			
No	239 (97.2)	513 (90.5)	0.0007
Yes	7 (2.8)	54 (9.5)	
**Outcome in the first 24 hours**			
Discharge	204 (82.9)	349 (61.6)	<0.0001
Admission to a room	20 (8.1)	35 (6.2)	
Admission to semi-intensive care unit	12 (4.9)	86 (15.2)	
Admission to ICU §	10 (4.1)	77 (13.6)	
Death	0 (-)	20 (3.5)	
**Final Outcome**			
Discharge	235 (95.5)	469 (82.7)	<0.0001
Death	11 (4.5)	98 (17.3)	

*n = Absolute number; †p = Significance level; ‡% = Percentage; §ICU = Intensive Care Unit

Regarding patient outcomes in the first 24 hours of admission to the ED, 553 (n= 68%) were discharged, 98 (n= 12.1%) were admitted to semi-intensive care, 87 (n= 10.7%) were admitted to the ICU, 55 (n= 6.8%) were admitted to a room and 20 (n= 2.5) succumbed to death.

When associated with the first clinical deterioration presented in the first 24 hours, it was found that patients with positive qSOFA on admission had a higher percentage of respiratory failure and CPA than patients with a negative score with a significance level (p) <0.0001 (Table 3).

**Table 3 -:** Association between quick Sequential Organ Failure Assessment (qSOFA*) and clinical deterioration on admission and immediately before the event (n^†^ = 187). São Paulo, SP, Brazil, 2022

qSOFA*	**ARF** ^‡^	**Shock**	**CPA** ^§^	**ALOC** ^║^		
	**n** ^†^ **(%** ^¶^ **)**	**n** ^†^ **(%** ^¶^ **)**	**n** ^†^ **(%** ^¶^ **)**	**n** ^†^ **(%** ^¶^ **)**	**Total**	**p** ** **value**
**On admission**						
Negative	89 (11.9)	5 (0.7)	5 (0.7)	21 (2.8)	120	<0.0001
Positive	47 (70.1)	1 (1.5)	17 (25.4)	2 (3)	67	
**Before deterioration**						
Negative	83 (11.5)	5 (0.7)	5 (0.7)	16 (2.2)	109	<0.0001
Positive	53 (59.6)	1 (1.1)	17 (19.1)	7 (7.9)	78	

*qSOFA = quick Sequential Organ Failure Assessment; †n = Absolute number; ‡ARF = Respiratory failure; §CPA = Cardiopulmonary arrest; ║ALOC = Altered level of consciousness; ¶% = Percentage; **p = Significance level

Patients with negative qSOFA on admission had a higher percentage of absence of clinical deterioration than patients with positive qSOFA (p< 0.0001).

Regarding the risk classification category on admission, patients who presented positive qSOFA had a higher percentage of ESI 2 than those with negative qSOFA (p< 0.0001), and patients with negative qSOFA had a higher percentage of ESI 3 and ESI 4 (p< 0.0001).

Furthermore, patients with negative qSOFA had a higher percentage of discharge than patients with positive qSOFA (p< 0.0001).

qSOFA also showed higher specificity and low sensitivity for tracking clinical deterioration, respiratory failure, shock, CPA, and altered level of consciousness (Table 4).

In simple logistic regression, the variable that best explained clinical deterioration was qSOFA. Participants with a positive qSOFA on admission were 350 times more likely to present deterioration compared to participants with a negative qSOFA. Participants with a positive qSOFA before the event were 40 times more likely to present deterioration compared to participants with a negative qSOFA. On the other hand, in the multiple logistic regression, the variable that best explained deterioration was the qSOFA on admission: patients with a positive qSOFA were 352 times more likely to deteriorate when compared to patients with a negative qSOFA (p< 0.0001 95% CI [48.46; 2562.12]).

**Table 4 -:** Sensitivity and specificity of quick Sequential Organ Failure Assessment (qSOFA*) for identifying clinical deterioration before the event (n† = 813). São Paulo, SP, Brazil, 2022

**Types of clinical deterioration**	**Clinical deterioration**			
	**No**	**Yes**	**Sensitivity**	**Specificity**
	**n** ^†^ **(%** ^‡^ **)**	**n** ^†^ **(%** ^‡^ **)**	**(%** ^‡^ **)**	**(%** ^‡^ **)**
**Clinical deterioration in the Emergency Department**				
Negative qSOFA *	615 (75.6)	109 (13.4)	41.7	98.2
Positive qSOFA *	11 (1.4)	78 (9.6)		
**Respiratory failure**				
Negative qSOFA *	627 (77.1)	97 (11.9)	39.0	95.9
Positive qSOFA *	27 (3.3)	62 (7.6)		
**Shock**				
Negative qSOFA *	696 (85.6)	28 (3.4)	6.7	88.9
Positive qSOFA *	87 (10.7)	2 (0.2)		
**Cardiopulmonary arrest**				
Negative qSOFA *	679 (83.5)	45 (5.5)	33.8	91.3
Positive qSOFA *	66 (8.1)	23 (2.8)		
**Altered level of consciousness**				
Negative qSOFA *	683 (84)	41 (5.0)	32.8	90.8
Positive qSOFA *	69 (8.5)	20 (2.5)		

*qSOFA = quick Sequential Organ Failure Assessment; †n = Absolute number; ‡% = Percentage

**Figure 1 -:**
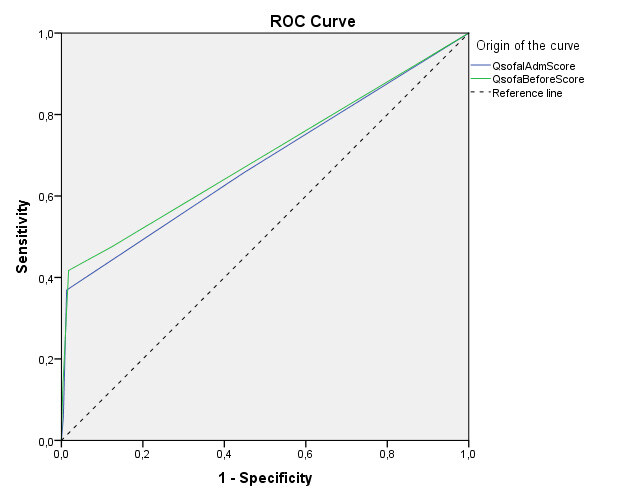
ROC curve of qSOFA on admission and immediately before clinical deterioration

When analyzing the performance of qSOFA to identify deterioration, the area under the ROC curve was 0.68 (0.63–0.73) on patient admission, and 0.70 (0.65–0.75) immediately before the event, as shown in Figure 1.

According to ROC curve analysis, the cutoff point for clinical deterioration was ≥ 1, both on admission and immediately before clinical deterioration. On admission, qSOFA had 66% sensitivity and 55% specificity, and immediately before clinical deterioration, 48% sensitivity and 88% specificity.

## Discussion

In this study, we evaluated the performance of qSOFA as a predictive tool for clinical deterioration in patients with COVID-19 who were treated in an ED. The use of early warning scores has been gaining prominence in the screening of patients at risk of clinical deterioration^(14)^, and their use seems reasonable, since the WHO has issued warnings about the risks of future pandemics^(15)^. qSOFA was developed and recommended as a clinical assessment system to stratify patients based on sepsis severity^(16)^. A qSOFA score of 2 or more may indicate an unfavorable prognosis^(17)^.

The average age in this study was higher than the global average^(18)^. Most of our patients were classified in categories ESI 3 and ESI 2, which indicate that the patient must be seen within 15 minutes, as they are considered very urgent or require some resource^(4)^. Few studies have associated triage systems with outcomes or have been validated for this scenario. A retrospective study, which used the Manchester Triage System and was developed in southern Brazil with 1228 patients, had similar findings, that is, most patients with COVID-19 were classified as very urgent and urgent^(19)^. This fact may be associated with the lack of seeking medical care in the presence of mild symptoms, in accordance with the health guidelines at that time^(20)^.

The Centers for Disease Control and Prevention (CDC) issued a report from the first months of the pandemic on 1.3 million reported cases, indicating a mortality rate of around 2.5%, with the majority of those infected being oligosymptomatic or asymptomatic. However, approximately 14% of the population experienced more acute symptoms of viral pneumonia, and 2% could develop the severe form of the disease, requiring intensive care^(21)^. Most of the patients who sought emergency care were discharged, but we observed a relationship between age and comorbidities with negative outcomes. However, more than twice as many were hospitalized, and of these, 41% were admitted to the ICU or died within the first 24 hours. These differences can be explained, in part, by the fact that the average age of our population was higher than the global average, in addition to the fact that almost 70% of patients had at least one comorbidity, especially cardiovascular. The relationship between age group, presence of comorbidities and severity of the disease is already well established. A characterization study showed that octogenarians were 20 times more likely to die when compared to the age group of 50-59 years^(22)^. Another study demonstrated 6 times more hospitalizations and 12 times more deaths among those with comorbidities^(21)^.

The period of greatest severity of the disease generally begins after the 6^th^ day of the onset of symptoms, since the peak of the viral load in the host is expected to have been reached^(23-24)^, and this is when most patients present to the ED with signs of viral pneumonia. Most of our patients sought care within the first 6 days of symptoms, which draws our attention to the possibility of clinical deterioration in the following days of hospitalization, since the development of Acute Respiratory Distress Syndrome occurs between the 7^th^ and 9^th^ days^(24)^. In fact, among the 260 hospitalized patients, 187 presented some type of clinical deterioration in the first 24 hours, with respiratory failure being the most prevalent condition, occurring in 72.7% of cases of clinical deterioration. SARS-CoV2, upon reaching the lower airways, infects especially T2 pneumocytes (cells that express angiotensin-converting enzyme 2 in large quantities), leading to viral pneumonia and, in some cases, shock secondary to Systemic Inflammatory Response Syndrome (SIRS)^(24)^. Several reports indicate respiratory failure as the most prevalent clinical deterioration in patients with COVID-19^(2,24-25)^.

In an emergency room setting, especially during peak periods and overload, both for patients being treated and for those hospitalized waiting for a bed, situations of insufficient monitoring may occur. In this context, it becomes opportune to implement tracking and emergency response systems^(26)^. Several screening systems were adapted to include the typical characteristics of the disease: signs of respiratory failure^(3,27-28)^.

qSOFA is one such tool, validated in the field for tracking clinical signs of sepsis. Since it is a simple score applied at the bedside, which in practice facilitates adherence, its validation in different scenarios, such as in tracking respiratory failure due to viral pneumonia, seems appropriate to us^(17)^.

In this case study, positive qSOFA was associated with ESI 2, a higher rate of clinical deterioration, ICU admission and mortality. qSOFA showed high specificity but low sensitivity for all types of clinical deterioration. Patients with a positive qSOFA on admission were 350 times more likely to have some clinical deterioration compared to those with a negative qSOFA. In the ROC curve, the cutoff point for clinical deterioration was ≥ 1, both on admission and immediately before the event, with better sensitivity (66%) being found at admission, and specificity (88%) immediately before the event.

Our findings, when contextualized, make sense, since qSOFA has the respiratory rate count as the only early pattern of respiratory failure, the most prevalent clinical deterioration in our population and in the literature^(3,27-28)^.

Regarding performance, a systematic review analyzed the performance of several scores with mortality, and qSOFA scored 0.622 on the ROC curve, showing lower performance; however, it did not analyze it in terms of clinical deterioration^(29)^. We draw attention to the real purpose of early warning scores which, when designed and developed, have the function of tracking clinical worsening, and not predicting mortality^(14)^. In our study, qSOFA obtained 0.68 (95%; 0.63-0.73) at admission, and immediately before the event it was higher, 0.70 (95%; 0.65-0.75), which can be considered a useful tool, especially when the cutoff point is reduced.

Apparently, qSOFA served as a practical score with good results for emergency use in patients with COVID-19, being a clinically useful test according to the results of the area under the ROC curve.

This study is important for clinical practice, as it aims to increase health safety by identifying a clinical deterioration score for use in EDs, which have a global overcrowding scenario. In this study, qSOFA had high specificity but low sensitivity in patients with COVID-19, and should not be used alone.

In order to maximize safety, the importance of systematizing the assessment through early warning scores is highlighted. They are easy to apply, with data that are routinely measured in all patients. The findings of this study can help professionals create strategies to organize the flow of care, in order to improve the care process, not only in the ED, but also in inpatient units.

This study had the limitation of portraying the reality of a single center of a philanthropic institution, with a population that is predominantly private medicine, which may restrict the generalization of the results.

## Conclusion

qSOFA demonstrated specificity higher than 98%; however, it demonstrated low sensitivity for detecting clinical deterioration. This indicates that it should not be used alone in clinical practice, but it has the potential to complement the clinical assessment of adult patients with COVID-19 in the emergency department.
