# Supplementary material for: Quick Sequential Organ Failure Assessment to identify clinical deterioration in adults with COVID-19: a retrospective cohort
Source: Rev Lat Am Enfermagem. 2025 May 19;33:e4530. doi: 10.1590/1518-8345.7239.4530 (PMC12088658; doi:10.1590/1518-8345.7239.4530)
Supplement: Translation

# Translation: Quick Sequential Organ Failure Assessment para identificar deterioração clínica em adultos com COVID-19: coorte retrospectiva*

**DOI:** 10.1590/1518-8345.7239.4531

**Authors:** Luiz Felipe Sales Mauricio, Cassia Regina Vancini Campanharo, Luiz Humberto Vieri Piacezzi, Maria Carolina Barbosa Teixeira Lopes, Ruth Ester Assayag Batista

**Affiliations:** 1 Universidade Federal de São Paulo, Escola Paulista de Enfermagem, São Paulo, SP, Brasil

**Keywords:** COVID-19, Sinais Vitais, Escore de Alerta Precoce, Deterioração Clínica, Cuidado de Enfermagem, Serviço Hospitalar de Emergência

## Abstract

avaliar o desempenho do qSOFA na identificação de deterioração em pacientes com COVID-19.

coorte retrospectiva, realizada entre fevereiro e agosto de 2020, no Serviço de Emergência de um hospital privado, com 813 adultos. Foram estudadas as variáveis: sociodemográficas, clínicas, deterioração, qSOFA na admissão e antes do evento, e desfechos. O desempenho do qSOFA nos dois momentos foi analisado pela área sob a curva ROC.

a média de idade foi de 69 anos. Predominaram homens (61,5%), brancos (97,2%), católicos (73,7%), casados (89,6%) e assalariados (66%). Comorbidades estavam presentes em 69,7%, e 58,8% foram classificados como “urgentes” na admissão. A deterioração mais frequente foi insuficiência respiratória (16,7%), e o desfecho, alta (68%). Pacientes com qSOFA positivo na admissão apresentaram maior percentual de insuficiência respiratória, parada cardiorrespiratória e classificação de risco “muito urgente”, e os com qSOFA negativo, maior percentual de alta (p<0,0001). Na admissão, o qSOFA teve 66% de sensibilidade e 55% de especificidade, e antes do evento, 48% de sensibilidade e 88% de especificidade para identificação de deterioração. Pacientes com qSOFA positivo, na admissão, apresentaram 350 vezes mais chance de deterioração.

o qSOFA apresentou sensibilidade baixa para identificação de deterioração nos dois momentos e especificidade alta antes do evento.

## Destaques

**(1)** Pacientes com COVID-19 e qSOFA positivo apresentaram maior risco de morte. **(2)** O qSOFA positivo detectou precocemente a deterioração clínica em pacientes com COVID-19. **(3)** Pacientes com COVID-19 e qSOFA positivo utilizaram mais recursos diagnósticos.

## Introdução

O vírus SARS-COV-2, responsável pela doença COVID-19, foi identificado na República Popular da China no fim de 2019^(1)^, espalhando-se rapidamente pelo mundo^(2-3)^, e em março de 2020 a Organização Mundial da Saúde (OMS) declarou a pandemia^(1)^. Até dezembro de 2022, já haviam sido confirmados 662.413.265 casos e 6.687.694 mortes causadas pela COVID-19 no mundo^(4)^. No Brasil, no mesmo período, havia 36.202.186 casos confirmados e 693.017 óbitos^(4)^.

A COVID-19 possui perfis clínicos distintos, desde uma infecção assintomática até quadros clínicos críticos. Os sinais e sintomas mais frequentes são febre, cansaço e tosse seca; entretanto, alguns casos podem evoluir para insuficiência respiratória, com necessidade de suporte ventilatório, choque séptico/sepse, insuficiência renal, tromboembolismo e parada cardiorrespiratória (PCR)^(2,4)^, o que torna obrigatória a identificação precoce de sinais de deterioração clínica.

Nesse contexto, a avaliação da condição clínica do paciente é de extrema importância, e deve ter início na classificação de risco para o gerenciamento do fluxo de atendimento correto e atendimento imediato em condições nas quais há risco de morte^(2)^.

Não obstante, escores de identificação precoce de deterioração clínica, que é definida como a piora do estado clínico de um indivíduo, manifestada por alterações nos sinais vitais, no estado mental, ou em outros parâmetros clínicos, que indicam potencial ameaça à vida ou a necessidade de uma intervenção médica urgente^(5)^, têm sido utilizados com o objetivo de complementar a avaliação clínica no serviço de emergência. Identificar um escore acurado para rastrear a deterioração clínica pode melhorar a qualidade e a segurança da assistência. O *quick Sequential Organ Failure Assessment* (qSOFA) tem sido um escore recomendado para identificar pacientes com infecção com alto risco de ter desfechos ruins em cenários externos à unidade de terapia intensiva (UTI)^(6)^. Resumido do *Sequential Organ Failure Assessment* (SOFA)*,* um consagrado escore para estratificar risco em pacientes com sepse, o qSOFA é uma ferramenta de alerta simples que pode ser usado em qualquer cenário, com o objetivo de rastrear sinais clínicos de deterioração relacionados à sepse^(7)^_._ Com a análise de apenas 3 sinais clínicos – frequência respiratória, pressão arterial sistólica e status mental –, o qSOFA apresentou bom desempenho em cenário de sepse^(8)^. Em uma metanálise que incluiu seis estudos com 17.868 pacientes, o qSOFA foi considerado um bom preditor de mortalidade por pneumonia^(8)^. Em outra revisão, o qSOFA apresentou bom desempenho em prever mortalidade em pacientes com ou sem sinais infecciosos^(9)^.

Como na COVID-19 a pneumonia é a principal complicação e pode cursar, rapidamente, para a insuficiência respiratória, o qSOFA pode ser útil para identificar, de forma simples e precoce, a piora clínica. A avaliação de forma rápida e sistematizada detectará sinais de instabilidade clínica, possibilitando a intervenção rápida com consequente aumento da sobrevida dos pacientes^(6)^. Apesar de não terem sido encontrados estudos que recomendem um sistema de alerta precoce (EWS) específico para os pacientes com COVID-19, é razoável supor que a sua utilização pode melhorar a qualidade da documentação dos parâmetros vitais, auxiliando nas avaliações sistemáticas e recorrentes dos enfermeiros.

Não somente durante a pandemia de COVID-19, os serviços de emergências têm, há mais de uma década, a superlotação como um dos principais desafios para a manutenção da qualidade e da segurança assistencial. Diante da demanda aumentada de pacientes com quadros clínicos graves, com risco de morte, emerge a necessidade de identificar preditores precoces da piora clínica, de maneira a diminuir a mortalidade com implantação de terapêutica adequada em tempo oportuno. Contudo, pesquisas de enfermagem sobre a temática ainda são incipientes.

Por fim, avaliar qual escore tem melhor desempenho em um determinado cenário nos parece pertinente. Deste modo, esta casuística objetiva avaliar o desempenho do qSOFA em prever deterioração clínica em pacientes internados com COVID-19.

## Método

O relato metodológico deste estudo foi realizado conforme as diretrizes do *Strengthening the Reporting of Observational Studies in Epidemiology* (STROBE)^(10)^.

### Desenho e período do estudo

Coorte retrospectiva, realizada no período entre 1º de fevereiro a 3 de agosto de 2020, momento do primeiro pico da pandemia no Brasil, no Serviço de Emergência (SE) de um hospital privado no Sudeste do Brasil.

### População

Foram incluídos todos os pacientes admitidos no SE durante o período do estudo, com os seguintes critérios: maiores de 18 anos, com diagnóstico de COVID-19 e com completude das variáveis de interesse. Devido às características clínicas distintas, foram excluídos pacientes em cuidados paliativos, pois a coleta de dados envolveu variáveis que continham intervenções médicas ou de enfermagem com o objetivo de cura da doença ou prolongamento da vida do paciente; e gestantes, para as quais, por passarem por alterações fisiológicas específicas da gravidez, o qSOFA não tem validação robusta.

### Coleta de dados

O Serviço de Epidemiologia do hospital forneceu uma planilha do programa *MS Excel* ^®^ com a lista de todos os números de prontuários dos pacientes admitidos no SE com diagnóstico de COVID-19 no período de estudo. Em posse dessa lista, como o país continuava em pandemia, foi enviado por *e-mail* o Termo de Consentimento Livre e Esclarecido (TCLE). Os participantes foram incluídos na pesquisa após expressarem essa vontade por meio da assinatura eletrônica do TCLE. Em casos de pacientes que evoluíram a óbito, ou que não retornaram após três tentativas de contato e que não faziam seguimento na instituição, foi obtida dispensa para a aplicação do TCLE. Após a autorização dos participantes da pesquisa, foram acessados os prontuários eletrônicos, de setembro de 2021 a fevereiro de 2022, e os dados foram coletados pelo pesquisador principal e armazenados na plataforma *Research Electronic Data Capture* (*REDCap* ^®^)^(11)^, conforme recomendação da instituição. A segurança, o sigilo e a confidencialidade dos dados foram preservados, através da identificação dos pacientes por meio de códigos no banco de dados.

### Variáveis do estudo

Todos os dados dos pacientes foram extraídos exclusivamente dos prontuários eletrônicos dos participantes da pesquisa. As variáveis sociodemográficas coletadas foram: idade, gênero, etnia, religião e situação conjugal. As variáveis clínicas foram: tempo de início dos sintomas até procurarem atendimento médico, presença de comorbidade, categoria da classificação de risco, nível de consciência, sinais e sintomas.

O desfecho principal deste estudo foi a deterioração clínica, definida como a necessidade documentada de intervenção para restabelecer a homeostase, incluindo: introdução ou aumento do aporte de oxigênio, escalonamento na modalidade ventilatória, introdução de uma nova droga vasoativa ou ressuscitação cardiopulmonar. Ainda, foi registrado se o paciente apresentou ou não qualquer uma das deteriorações clínicas nas primeiras 24 horas.

Os desfechos secundários foram: alta, internação em apartamento, internação em unidade semi-intensiva, internação em unidade de terapia intensiva (UTI) e óbito nas primeiras 24 horas. E os desfechos finais foram: alta e óbito.

A instituição onde o estudo foi realizado utiliza como sistema de classificação de risco no SE o *Emergency Severity Index* (Índice de Gravidade de Emergência - ESI). Nesse protocolo, as categorias da classificação de risco são: 1, 2, 3, 4 e 5. Os níveis 1 e 2 correspondem aos pacientes que estão em situações de emergência, ou seja, com risco de morte; nos níveis 3, 4 e 5 os pacientes são classificados pela necessidade de recursos, como por exemplo, exames diagnósticos complementares, procedimentos terapêuticos a serem utilizados e frequência de avaliação dos sinais vitais. Pacientes na categoria 3 são classificados como urgente, na 4 como pouco urgente, e na 5 como não urgente^(12)^.

O qSOFA foi calculado em dois momentos: na admissão no SE, durante a classificação de risco realizada pelo enfermeiro, que avalia e insere os valores no prontuário eletrônico, sendo o cálculo realizado automaticamente pelo sistema; e em caso de deterioração clínica (DC) nas primeiras 24 horas após a admissão no SE, sendo o cálculo realizado com base nos últimos parâmetros registrados no prontuário pelo profissional responsável pelo atendimento ao paciente. O qSOFA é composto pelas variáveis: frequência respiratória, nível de consciência e pressão arterial sistólica^(6)^. Deve ser atribuído um ponto se a frequência respiratória é maior que 22 incursões por minuto, se há alteração do nível de consciência (escore segundo a Escala de Coma de Glasgow inferior a 15), ou se a pressão arterial sistólica é menor de 100 mmHg^(6)^. Define-se como qSOFA positivo quando duas ou mais variáveis são pontuadas, e negativo quando uma ou nenhuma é pontuada^(6)^. A pontuação mais alta (3 pontos) está associada a mau prognóstico^(13)^. Quando o escore é ≥ 2, estudos apontam como indicador de potencial sepse em pacientes com suspeita de infecção fora da UTI^(13)^.

### Tratamento e análise dos dados

Os dados da pesquisa foram armazenados na plataforma de coleta *REDCap*®^(11)^ e foi utilizado o *Statistical Package for the Social Sciences* (SPSS), versão 23, para processar a análise. A análise descritiva foi realizada por meio dos cálculos de média, desvio-padrão, mediana, mínimo e máximo. Para as variáveis categóricas calculou-se frequência e percentual. A associação do qSOFA (positivo ou negativo) com a ocorrência de deterioração clínica, comorbidade, por categoria da classificação de risco, desfecho nas 24 horas da admissão e desfecho final foi verificada pelo teste de qui-quadrado, e, quando necessário, utilizou-se o teste exato de Fisher. O teste de Mann-Whitney ou teste de Kruskal-Wallis foi utilizado para 3 categorias ou mais. Foram calculadas a sensibilidade e a especificidade do qSOFA para ocorrência de deterioração clínica. Para todas as análises, foi considerado o valor de p< 0,05 para significância estatística. Foi realizada a regressão logística simples para verificar a relação das variáveis independentes em relação à deterioração clínica (variável dependente). A regressão logística múltipla foi realizada para verificar o fator que melhor explicava a “deterioração clínica”. O método de seleção utilizado foi o *Forward*. O desempenho do qSOFA para identificação de deterioração clínica na admissão e antes do evento foi analisado por meio da área sob a curva ROC.

### Aspectos éticos

Essa pesquisa está em conformidade com a Resolução 466/2012 do Conselho Nacional de Saúde e foi aprovada pelo Comitê de Ética em Pesquisa da instituição, parecer 4.919.847, Certificado de Apresentação para Apreciação Ética (CAAE): 32684620.3.3001.0071, e pelo Sistema Gerenciador de Projetos de Pesquisa da instituição coparticipante, sob parecer n° 4316-20.

## Resultados

Nesta pesquisa não houve perdas de seguimento. Foram incluídos 813 pacientes, com média de idade de 69 anos (± 17,8), e com prevalência do sexo masculino (61,5%, n= 500), brancos (97,2%, n= 769), católicos (73,7%, n= 583), casados (89,6%, n= 578) e assalariados (66%, n= 456). A maioria da população do estudo (75,3%, n= 612) procurou atendimento médico entre o primeiro e o sexto dia do início dos sintomas, 21,6% (n= 176) entre o sétimo e o décimo, e 3,1% (n= 25) com mais de 10 dias de sintomas.

Com relação à classificação de risco, do total de 813 (n= 100%) pacientes, 58,8% (n= 478) foram classificados como ESI 3, 21,9% (n= 178) ESI 2, 18% (n= 146) ESI 4 e 1,4% (n= 11) ESI 1.

Quanto à ocorrência de deterioração clínica, 16,7% (n= 136) dos indivíduos apresentaram insuficiência respiratória aguda nas primeiras 24 horas da admissão no SE, 0,7% (n=6) choque, 2,7% (n=22) PCR e 2,8% (n=23) alteração do nível de consciência. Ocorreram 187 deteriorações clínicas nas primeiras 24 horas da admissão no SE, de acordo com os dados da Tabela 1.

**Tabela 1 -:** Deterioração clínica dos pacientes com COVID-19 nas primeiras 24 horas da admissão no Serviço de Emergência (n* = 813). São Paulo, SP, Brasil, 2022

**Deterioração clínica**	**24 horas da admissão**
	n* (% ^†^ **)**
**Insuficiência respiratória aguda**	
Não	677 (83,3)
Sim	136 (16,7)
**Choque**	
Não	807 (99,3)
Sim	6 (0,7)
**Parada cardiorrespiratória**	
Não	791 (97,3)
Sim	22 (2,7)
**Alteração do nível de consciência**	
Não	790 (97,2)
Sim	23 (2,8)

*n = Número absoluto; ^†^% = Percentual

Da população estudada (n= 813), 69,7% (n= 567) possuíam alguma comorbidade, sendo 41% (n= 332) cardiovasculares, 34,4% (n= 280) respiratórias, 20,4% (n= 166) endócrinas, e 10,8% (n= 88) neurológicas.

Indivíduos portadores de comorbidades apresentaram maior percentual de insuficiência respiratória, PCR e alteração do nível de consciência do que aqueles sem comorbidades (p< 0,0001). Dos indivíduos que possuíam algum tipo de comorbidade, 106 (n= 18,7%) apresentaram insuficiência respiratória, 6 (n= 1,1%) choque, 22 (n= 3,9%) parada cardiorrespiratória, e 20 (n= 3,5%) alteração do nível de consciência. Foi observado que os pacientes portadores de alguma comorbidade apresentaram maior percentual de deterioração clínica do que aqueles sem (p< 0,0001), de insuficiência respiratória aguda (p= 0,0019), choque (p= 0,0036), PCR (p= 0,0083), e alteração do nível de consciência (p= 0,0007), quando comparados aos que não tinham (p< 0,0001). Os pacientes com comorbidades tiveram maior percentual de internação em semi-intensiva (p< 0,0001) e UTI (p< 0,0001), e maior frequência de óbito (p< 0,0001). Indivíduos sem comorbidades tiveram mais alta hospitalar quando comparados com aqueles com comorbidades (p< 0,0001) (Tabela 2).

**Tabela 2 -:** Associação da presença de comorbidades com as deteriorações clínicas e os desfechos na população estudada (n* = 813). São Paulo, SP, Brasil, 2022

Variáveis	**Presença de comorbidades**		**p** ^†^ **-valor**
	Não n* (% ^‡^ **)**	Sim n* (% ^‡^ **)**	
**Deterioração clínica na admissão**			
Não	213 (86,6)	413 (72,8)	<0,0001
Sim	33 (13,4)	154 (27,2)	
**Insuficiência respiratória**			
Não	214 (87)	440 (77,6)	0,0019
Sim	32 (13)	127 (22,4)	
**Choque**			
Não	244 (99,2)	539 (95,1)	0,0036
Sim	2 (0,8)	28 (4,9)	
**Parada cardiorrespiratória**			
Não	235 (95,5)	510 (89,9)	0,0083
Sim	11 (4,5)	57 (10,1)	
**Alteração do nível de consciência**			
Não	239 (97,2)	513 (90,5)	0,0007
Sim	7 (2,8)	54 (9,5)	
**Desfecho nas primeiras 24 horas**			
Alta	204 (82,9)	349 (61,6)	<0,0001
Internação em Apartamento	20 (8,1)	35 (6,2)	
Internação em Semi-intensiva	12 (4,9)	86 (15,2)	
Internação em UTI ^§^	10 (4,1)	77 (13,6)	
Óbito	0 (-)	20 (3,5)	
**Desfecho Final**			
Alta	235 (95,5)	469 (82,7)	<0,0001
Óbito	11 (4,5)	98 (17,3)	

*n = Número absoluto; ^†^p = Nível de significância; ^‡^% = Percentual; ^§^UTI = Unidade de Terapia Intensiva

Com relação aos desfechos dos pacientes nas primeiras 24 horas de admissão no SE, 553 (n= 68%) tiveram alta, 98 (n= 12,1%) foram internados em semi-intensiva, 87 (n= 10,7%) foram internados em UTI, 55 (n= 6,8%) internados em apartamento, e 20 (n= 2,5) evoluíram a óbito.

Quando associado à primeira deterioração clínica apresentada nas primeiras 24 horas, verificou-se que pacientes com qSOFA positivo na admissão apresentaram maior percentual de insuficiência respiratória e PCR do que pacientes com escore negativo, com nível de significância (p) < 0,0001 (Tabela 3).

**Tabela 3 -:** Associação entre o *quick Sequential Organ Failure Assessment* (qSOFA*) e deterioração clínica na admissão e imediatamente antes do evento (n^†^ = 187). São Paulo, SP, Brasil, 2022

qSOFA*	**IRP** ^‡^	**Choque**	**PCR** ^§^	**ANC** ^║^		
	**n** ^†^ **(%** ^¶^ **)**	**n** ^†^ **(%** ^¶^ **)**	**n** ^†^ **(%** ^¶^ **)**	**n** ^†^ **(%** ^¶^ **)**	**Total**	**p** ** **valor**
**Na admissão**						
Negativo	89 (11,9)	5 (0,7)	5 (0,7)	21 (2,8)	120	<0,0001
Positivo	47 (70,1)	1 (1,5)	17 (25,4)	2 (3)	67	
**Antes da deterioração**						
Negativo	83 (11,5)	5 (0,7)	5 (0,7)	16 (2,2)	109	<0,0001
Positivo	53 (59,6)	1 (1,1)	17 (19,1)	7 (7,9)	78	

*qSOFA = *quick Sequential Organ Failure Assessment*; ^†^n = Número absoluto; ^‡^IRP = Insuficiência respiratória; ^§^PCR = Parada cardiorrespiratória; ^║^ANC = Alteração do nível de consciência; ^¶^% = Percentual; **p = Nível de significância

Pacientes com qSOFA negativo na admissão apresentaram maior percentual de ausência de deterioração clínica do que pacientes com qSOFA positivo (p< 0,0001).

Com relação à categoria de classificação de risco na admissão, pacientes que apresentaram qSOFA positivo tiveram maior percentual de ESI 2 do que aqueles com qSOFA negativo (p< 0,0001), e pacientes com qSOFA negativo apresentaram maior percentual de ESI 3 e ESI 4 (p< 0,0001).

Além disso, os pacientes com qSOFA negativo apresentaram maior percentual de alta do que pacientes com qSOFA positivo (p< 0,0001).

O qSOFA também apresentou maior especificidade e baixa sensibilidade para rastrear deterioração clínica, insuficiência respiratória, choque, PCR e alteração do nível de consciência (Tabela 4).

Na regressão logística simples, a variável que melhor explicou a deterioração clinica foi o qSOFA. Os participantes com qSOFA positivo na admissão apresentaram 350 vezes mais chances de sofrer deterioração em relação aos participantes com qSOFA negativo. Os participantes com qSOFA positivo antes do evento apresentaram 40 vezes mais chances de sofrer deterioração em relação aos participantes com qSOFA negativo. Por outro lado, na regressão logística múltipla, a variável que melhor explicou a deterioração foi o qSOFA na admissão: pacientes com qSOFA positivo apresentaram 352 vezes mais chance de deterioração quando comparados aos pacientes com qSOFA negativo (p< 0,0001 IC de 95% [48,46; 2562,12]).

**Tabela 4 -:** Sensibilidade e especificidade do *quick Sequential Organ Failure Assessment* (qSOFA*) para identificação da deterioração clínica antes do evento (n^†^ = 813). São Paulo, SP, Brasil, 2022

**Tipos de deterioração clínica**	**Deterioração clínica**			
	**Não**	**Sim**	**Sensibilidade**	**Especificidade**
	**n** ^†^ **(%** ^‡^ **)**	**n** ^†^ **(%** ^‡^ **)**	**(%** ^‡^ **)**	**(%** ^‡^ **)**
**Deterioração clínica no Serviço de Emergência**				
qSOFA * Negativo	615 (75,6)	109 (13,4)	41,7	98,2
qSOFA * Positivo	11 (1,4)	78 (9,6)		
**Insuficiência respiratória**				
qSOFA * Negativo	627 (77,1)	97 (11,9)	39,0	95,9
qSOFA * Positivo	27 (3,3)	62 (7,6)		
**Choque**				
qSOFA * Negativo	696 (85,6)	28 (3,4)	6,7	88,9
qSOFA * Positivo	87 (10,7)	2 (0,2)		
**Parada cardiorrespiratória**				
qSOFA * Negativo	679 (83,5)	45 (5,5)	33,8	91,3
qSOFA * Positivo	66 (8,1)	23 (2,8)		
**Alteração do nível de consciência**				
qSOFA * Negativo	683 (84)	41 (5,0)	32,8	90,8
qSOFA * Positivo	69 (8,5)	20 (2,5)		

*qSOFA = *quick Sequential Organ Failure Assessment*; ^†^n = Número absoluto; ^‡^% = Percentual

Ao analisar o desempenho do qSOFA para identificar deterioração, a área sob a curva ROC foi 0,68 (0,63 – 0,73) na admissão do paciente, e 0,70 (0,65 – 0,75) imediatamente antes do evento, conforme apresentado na Figura 1.

**Figura 1 -:** Curva ROC do qSOFA na admissão e imediatamente antes da deterioração clínica

De acordo com a análise da curva ROC, o ponto de corte para deterioração clínica foi ≥ 1, tanto na admissão quanto imediatamente antes da deterioração clínica. Na admissão, o qSOFA teve 66% de sensibilidade e 55% de especificidade, e imediatamente antes da deterioração clínica, 48% de sensibilidade e 88% de especificidade.

## Discussão

Neste estudo, avaliamos o desempenho do qSOFA como ferramenta preditiva de deterioração clínica em pacientes com COVID-19 que foram atendidos em um SE. O uso de escores de alerta precoce vem ganhando protagonismo no rastreio de pacientes com risco de deterioração clínica^(14)^, e o seu uso parece racional, uma vez que a OMS emitiu alertas para os riscos de pandemias futuras^(15)^. O qSOFA foi desenvolvido e recomendado como um sistema de avaliação clínica para estratificar os pacientes com base na severidade da sepse^(16)^. Um escore qSOFA de 2 ou mais pode indicar um prognóstico desfavorável^(17)^.

A idade média neste estudo foi mais alta que a média global^(18)^. A maioria dos nossos pacientes foram classificados nas categorias ESI 3 e ESI 2, que indicam que o paciente deve ser atendido em até 15 minutos, por ser considerado muito urgente ou por necessitar utilizar algum recurso^(4)^. Poucos estudos associaram sistemas de triagem com desfechos ou foram validados para este cenário. Um estudo retrospectivo, que usou o Sistema de Triagem Manchester e foi desenvolvido no sul do Brasil com 1228 pacientes, teve achados semelhantes, ou seja, a maioria dos pacientes com COVID-19 foram classificados como muito urgentes e urgentes^(19)^. Esse fato pode estar associado à não procura por atendimento médico na presença de sintomas leves, conforme orientações sanitárias na época^(20)^.

O *Centers for Disease Control and Prevention* emitiu um relatório dos primeiros meses da pandemia sobre 1,3 milhões de casos reportados, indicando uma taxa de mortalidade em torno de 2,5%, sendo que a maioria dos infectados eram oligossintomáticos ou assintomáticos. Entretanto, uma parcela aproximada de 14% da população experenciou sintomas mais agudos de pneumonia viral, e 2% poderiam desenvolver a forma grave da doença, necessitando de cuidados intensivos^(21)^. Grande parte dos pacientes que procuraram o pronto-socorro foi dispensada, mas observamos uma relação entre idade e comorbidades com desfechos negativos. No entanto, mais que o dobro foram internados e, entre esses, 41% foram admitidos em UTI ou evoluíram para óbito nas primeiras 24 horas. Essas diferenças podem ser explicadas, em parte, pelo fato de a idade média da nossa população ter sido maior que a média global, além de quase 70% apresentarem ao menos uma comorbidade, especialmente cardiovasculares. A relação entre faixa etária, presença de comorbidades e a gravidade da doença já está bem estabelecida. Um estudo de caracterização apontou que octogenários tinham 20 vezes mais chances de óbito quando comparados à faixa etária de 50-59 anos^(22)^. Outro estudo demonstrou 6 vezes mais hospitalizações e 12 vezes mais mortes entre aqueles com comorbidades^(21)^.

O período de maior gravidade da doença geralmente se inicia após o 6° dia do início dos sintomas, uma vez que se espera ter atingido o pico da carga viral no hospedeiro^(23-24)^, e é quando a maioria dos pacientes se apresentam no SE com sinais de pneumonia viral. A maioria dos nossos pacientes buscaram atendimento nos primeiros 6 dias de sintomas, o que nos chama atenção para a possibilidade de deterioração clínica nos próximos dias de sua internação, uma vez que o desenvolvimento da Síndrome do Desconforto Respiratório Agudo ocorre entre o 7° e 9° dia^(24)^. De fato, entre os 260 pacientes internados, 187 pacientes apresentaram algum tipo de deterioração clínica nas primeiras 24 horas, sendo a insuficiência respiratória a condição mais prevalente, ocorrendo em 72,7% dos casos de deterioração clínica. O SARS-CoV2, ao atingir as vias aéreas inferiores, infecta especialmente os pneumócitos T2 (células que expressam enzima conversora de angiotensina 2 em grande quantidade), levando ao quadro de pneumonia viral e, em alguns casos, choque secundário à Síndrome da Resposta Inflamatória Sistêmica (SIRS)^(24)^. Diversos relatórios apontam a insuficiência respiratória como a deterioração clínica mais prevalente nos pacientes com COVID-19^(2,24-25)^.

Em um cenário de pronto-socorro, especialmente durante períodos de pico e sobrecarga, tanto para os pacientes em atendimento quanto para aqueles internados aguardando leito, podem ocorrer situações de monitorização insuficiente. Nesse contexto, torna-se oportuno implementar sistemas de rastreamento e resposta a emergências^(26)^. Diversos sistemas de triagem foram adaptados para que contemplassem a característica típica da doença: sinais de insuficiência respiratória^(3,27-28)^.

O qSOFA é uma dessas ferramentas, validado em campo para o rastreamento de sinais clínicos de sepse. Por se tratar de um escore simples e aplicado à beira do leito, o que na prática facilita a adesão, sua validação em cenários distintos, como no rastreamento da insuficiência respiratória por pneumonia viral, nos parece oportuno^(17)^.

Nesta casuística, o qSOFA positivo se associou à ESI 2, à maior taxa de deterioração clínica, à internação em UTI e à mortalidade. O qSOFA apresentou alta especificidade, mas baixa sensibilidade para todos os tipos de deterioração clínica. Pacientes com qSOFA positivo na admissão tinham 350 vezes mais chances de apresentarem alguma deterioração clínica, se comparados àqueles com o qSOFA negativo. Na curva ROC, o ponto de corte para deterioração clínica foi ≥ 1, tanto na admissão quanto imediatamente antes do evento, sendo que melhor sensibilidade (66%) foi encontrada na admissão, e especificidade (88%) imediatamente antes do evento.

Nossos achados, quando contextualizados, fazem sentido, uma vez que o qSOFA tem na contagem da frequência respiratória o único padrão precoce de insuficiência respiratória, a deterioração clínica mais prevalente em nossa população e na literatura^(3,27-28)^.

Com relação ao desempenho, uma revisão sistemática analisou a performance de diversos escores com a mortalidade, e o qSOFA pontuou 0,622 na curva ROC, mostrando menor desempenho; entretanto, não o analisou em termos de deterioração clínica^(29)^. Chamamos a atenção para o real propósito dos escores de alerta precoce que, quando idealizados e desenvolvidos, apresentam a função de rastreio da piora clínica, e não de prever a mortalidade^(14)^. Em nosso estudo, o qSOFA obteve 0,68 (95%;0,63-0,73) na admissão, e imediatamente antes do evento foi maior, 0,70 (95%;0,65-0,75), o que pode ser considerado uma ferramenta útil, especialmente quando reduzido o ponto de corte.

Aparentemente, o qSOFA serviu como um escore prático e com bons resultados para o uso na emergência em pacientes com COVID-19, sendo um teste clinicamente útil, conforme resultados da área sob a curva ROC.

Este estudo é importante para a prática clínica, pois tem como objetivo aumentar a segurança em saúde, por meio da identificação de um escore de deterioração clínica para uso em SE, que tem um cenário de superlotação mundialmente. Neste estudo, o qSOFA teve alta especificidade, mas baixa sensibilidade em pacientes com COVID-19, não devendo ser utilizado isoladamente.

Com o objetivo de maximizar a segurança, ressalta-se a importância da sistematização da avaliação, por meio de escores de alerta precoce. Eles são de fácil aplicabilidade, com dados que são rotineiramente mensurados em todos os pacientes. Os achados desde estudo podem ajudar os profissionais a criarem estratégias para organizar o fluxo de atendimento, a fim de melhorar o processo assistencial, não só nos SE, mas também nas unidades de internação.

Este estudo teve como limitação retratar a realidade de um único centro de uma instituição filantrópica, com clientela majoritariamente de medicina privada, o que pode restringir a generalização dos resultados.

## Conclusão

O qSOFA apresentou especificidade superior a 98%; entretanto, demonstrou baixa sensibilidade para rastrear a deterioração clínica. Isso indica que ele não deve ser utilizado de forma isolada na prática assistencial, mas possui potencial para compor a avaliação clínica dos pacientes adultos com COVID-19 no Serviço de Emergência.

# Translation: Quick Sequential Organ Failure Assessment para identificar el deterioro** clínico en adultos con COVID-19: cohorte retrospectiva*

**DOI:** 10.1590/1518-8345.7239.4529

**Authors:** Luiz Felipe Sales Mauricio, Cassia Regina Vancini Campanharo, Luiz Humberto Vieri Piacezzi, Maria Carolina Barbosa Teixeira Lopes, Ruth Ester Assayag Batista

**Affiliations:** 1 Universidade Federal de São Paulo, Escola Paulista de Enfermagem, São Paulo, SP, Brasil

**Keywords:** COVID-19, Signos Vitales, Puntuación de Alerta Temprana, Deterioro Clínico, Atención de Enfermería, Servicio de Urgencia en Hospital

## Abstract

evaluar el desempeño del qSOFA en la identificación de deterioro en pacientes con COVID-19.

cohorte retrospectiva, realizada entre febrero y agosto de 2020, en el Departamento de Emergencias de un hospital privado, con 813 adultos. Se estudiaron las siguientes variables: sociodemográficas, clínicas, de deterioro, qSOFA al ingreso y antes del evento, y desenlaces. El desempeño del qSOFA en ambos momentos se analizó utilizando el área bajo la curva ROC.

la edad promedio fue de 69 años. Predominaron los hombres (61,5%), blancos (97,2%), católicos (73,7%), casados (89,6%) y asalariados (66%). El 69,7% presentó comorbilidades y el 58,8% fueron clasificados como “urgentes” al ingreso. El deterioro más frecuente fue la insuficiencia respiratoria (16,7%), y el desenlace fue el alta (68%). Los pacientes con qSOFA positivo al ingreso tuvieron mayor porcentaje de insuficiencia respiratoria, paro cardiorrespiratorio y clasificación de riesgo “muy urgente”, y aquellos con qSOFA negativo tuvieron mayor porcentaje de alta (p< 0,0001). Al ingreso, el qSOFA tenía una sensibilidad del 66% y una especificidad del 55%, y antes del evento, una sensibilidad del 48% y una especificidad del 88% para identificar el deterioro. Los pacientes con qSOFA positivo al ingreso tenían 350 veces más probabilidades de deterioro.

el qSOFA mostró baja sensibilidad para identificar el deterioro en ambos momentos y alta especificidad antes del evento.

## Destacados

**(1)** Pacientes con COVID-19 y qSOFA positivo tenían mayor riesgo de muerte. **(2)** El qSOFA positivo detectó tempranamente deterioro clínico en pacientes con COVID-19. **(3)** Pacientes con COVID-19 y qSOFA positivo utilizaron más recursos diagnósticos.

## Introducción

El virus SARS-COV-2, responsable de la enfermedad COVID-19, fue identificado en la República Popular China a finales de 2019^(1)^, propagándose rápidamente por todo el mundo^(2-3)^, y en marzo de 2020 la Organización Mundial de la Salud (OMS) declaró la pandemia^(1)^. Hasta diciembre de 2022, se habían confirmado 662.413.265 casos y 6.687.694 muertes causadas por COVID-19 en el mundo^(4)^. En Brasil, en el mismo período, hubo 36.202.186 casos confirmados y 693.017 muertes^(4)^.

La COVID-19 tiene distintos perfiles clínicos, desde una infección asintomática hasta condiciones clínicas críticas. Los signos y síntomas más habituales son fiebre, cansancio y tos seca; sin embargo, algunos casos pueden progresar a insuficiencia respiratoria, requiriendo soporte ventilatorio, shock séptico/sepsis, insuficiencia renal, tromboembolismo y paro cardiorrespiratorio (PCR)^(2,4)^, lo que hace obligatoria la identificación temprana de signos de deterioro clínico.

En este contexto, la evaluación de la condición clínica del paciente es de suma importancia, debiendo comenzar con la clasificación de riesgo para garantizar una gestión adecuada del flujo de atención y una atención inmediata en situaciones en las que existe riesgo para la vida^(2)^.

Sin embargo, las puntuaciones de identificación temprana del deterioro clínico, que se define como el empeoramiento del estado clínico de un individuo, manifestado por cambios en los signos vitales, el estado mental u otros parámetros clínicos, que indican una amenaza potencial para la vida o la necesidad de una intervención médica urgente^(5)^, se han utilizado con el objetivo de complementar la evaluación clínica en el departamento de emergencias. Identificar una puntuación precisa para rastrear el deterioro clínico puede mejorar la calidad y la seguridad de la atención. El *quick Sequential Organ Failure Assessment* (qSOFA) ha sido una puntuación recomendada para identificar pacientes con infección con alto riesgo de tener malos desenlaces en entornos fuera de la unidad de cuidados intensivos (UCI)^(6)^. Resumido del *Sequential Organ Failure Assessment* (SOFA), una puntuación establecida para estratificar el riesgo en pacientes con sepsis, el qSOFA es una herramienta de alerta simple que se puede utilizar en cualquier escenario, con el objetivo de rastrear los signos clínicos de deterioro relacionados con la sepsis^(7)^_._ Con el análisis de solo tres signos clínicos – frecuencia respiratoria, presión arterial sistólica y estado mental –, el qSOFA tuvo un buen desempeño en el escenario de sepsis^(8)^. En un metaanálisis que incluyó seis estudios con 17.868 pacientes, el qSOFA se consideró un buen predictor de mortalidad por neumonía^(8)^. En otra revisión, el qSOFA tuvo buenos resultados en la predicción de la mortalidad en pacientes con o sin signos infecciosos^(9)^.

Como la neumonía es la principal complicación de la COVID-19 y puede conducir rápidamente a insuficiencia respiratoria, el qSOFA puede ser útil para identificar, de forma sencilla y temprana, el empeoramiento clínico. La evaluación rápida y sistemática detectará signos de inestabilidad clínica, permitiendo una intervención rápida con el consiguiente aumento de la supervivencia del paciente^(6)^. Aunque no se encontraron estudios que recomienden un sistema de alerta temprana (EWS) específico para pacientes con COVID-19, es razonable suponer que su uso puede mejorar la calidad de la documentación de parámetros vitales, ayudando en las evaluaciones sistemáticas y recurrentes de los enfermeros.

No solamente durante la pandemia de COVID-19, los servicios de emergencias llevan más de una década enfrentando la sobrecarga como uno de los principales desafíos para mantener la calidad y la seguridad asistencial. Dada la mayor demanda de pacientes con condiciones clínicas graves, en riesgo de muerte, existe la necesidad de identificar predictores tempranos de empeoramiento clínico, con el fin de reducir la mortalidad con la implementación de la terapia adecuada de manera oportuna. Sin embargo, las investigaciones de enfermería sobre el tema son aún incipientes.

Por último, nos parece pertinente evaluar qué puntuación se desempeña mejor en un escenario determinado. De este modo, esta casuística tiene como objetivo analizar el desempeño del qSOFA en la predicción del deterioro clínico en pacientes hospitalizados con COVID-19.

## Método

El informe metodológico de este estudio se llevó a cabo de acuerdo con las directrices de *Strengthening the Reporting of Observational Studies in Epidemiology* (STROBE)^(10)^.

### Diseño y periodo del estudio

Cohorte retrospectiva, realizada en el período comprendido entre el 1 de febrero y el 3 de agosto de 2020, momento del primer pico de la pandemia en Brasil, en el Departamento de Emergencias (DE) de un hospital privado del Sudeste de Brasil.

### Población

Se incluyeron todos los pacientes ingresados en el DE durante el período de estudio, con los siguientes criterios: mayores de 18 años, diagnosticados con COVID-19 y con completitud de las variables de interés. Por características clínicas distintas, fueron excluidos los pacientes que recibían cuidados paliativos, ya que la recolección de datos involucró variables que contenían intervenciones médicas o de enfermería con el objetivo de curar la enfermedad o prolongar la vida del paciente; y las mujeres embarazadas, para quienes, al sufrir cambios fisiológicos específicos del embarazo, el qSOFA no cuenta con una validación sólida.

### Recolección de datos

El Servicio de Epidemiología del hospital proporcionó una hoja de cálculo del programa *MS Excel* ^®^ con una lista de todos los números de registros médicos de los pacientes ingresados en el DE con diagnóstico de COVID-19 durante el período de estudio. En posesión de este listado, mientras el país permanecía en la pandemia, se envió por correo electrónico el Formulario de Consentimiento Libre e Informado (FCLI). Los participantes fueron incluidos en la investigación luego de expresar su deseo mediante la firma electrónica del FCLI. En los casos de pacientes que fallecieron, o que no regresaron después de tres intentos de contacto y que no fueron seguidos en la institución, se obtuvo una exención para la aplicación del FCLI. Después de la autorización de los participantes de la investigación, se accedió a los registros médicos electrónicos desde septiembre de 2021 hasta febrero de 2022, y los datos fueron recopilados por el investigador principal y almacenados en la plataforma *Research Electronic Data Capture* (*REDCap* ^®^)^(11)^, según lo recomendado por la institución. La seguridad, el secreto y la confidencialidad de los datos se preservaron por medio la identificación de los pacientes mediante códigos en la base de datos.

### Variables del estudio

Todos los datos de los pacientes se extrajeron exclusivamente de los registros médicos electrónicos de los participantes de la investigación. Las variables sociodemográficas recolectadas fueron: edad, género, etnia, religión y estado civil. Las variables clínicas fueron: tiempo desde el inicio de los síntomas hasta la búsqueda de atención médica, presencia de comorbilidad, categoría de clasificación de riesgo, nivel de conciencia, signos y síntomas.

El desenlace principal de este estudio fue el deterioro clínico, definido como la necesidad documentada de intervención para restablecer la homeostasis, que incluye: introducción o aumento del suministro de oxígeno, aumento del modo de ventilación, introducción de un nuevo fármaco vasoactivo o reanimación cardiopulmonar. Además, se registró si el paciente presentó o no algún deterioro clínico en las primeras 24 horas.

Los desenlaces secundarios fueron: alta, ingreso a un apartamento, ingreso a una unidad semiintensiva, ingreso a una unidad de cuidados intensivos (UCI) y muerte dentro de las primeras 24 horas. Y los desenlaces finales fueron: alta y muerte.

La institución donde se realizó el estudio utiliza el *Emergency Severity Index* (Índice de Severidad de Emergencias - ESI) como sistema de clasificación de riesgos en el DE. En este protocolo las categorías de clasificación de riesgo son: 1, 2, 3, 4 y 5. Los niveles 1 y 2 corresponden a pacientes que se encuentran en situaciones de emergencia, es decir, en riesgo de muerte; en los niveles 3, 4 y 5, los pacientes se clasifican según la necesidad de recursos, como exámenes diagnósticos complementarios, procedimientos terapéuticos a utilizar y frecuencia de evaluación de signos vitales. Los pacientes de la categoría 3 se clasifican como urgente, de la categoría 4 como ligeramente urgente y de la categoría 5 como no urgente^(12)^.

El qSOFA se calculó en dos momentos: al ingreso al DE, durante la clasificación de riesgo realizada por el enfermero, quien evalúa e inserta los valores en la historia clínica electrónica, siendo el cálculo realizado automáticamente por el sistema; y en caso de deterioro clínico (DC) en las primeras 24 horas posteriores al ingreso al DE, siendo el cálculo se realizado con base en los últimos parámetros registrados en la historia clínica por el profesional responsable de la atención del paciente. El qSOFA está compuesto por las variables: frecuencia respiratoria, nivel de conciencia y presión arterial sistólica^(6)^. Se debe asignar un punto si la frecuencia respiratoria es superior a 22 respiraciones por minuto, si hay un cambio en el nivel de conciencia (puntuación en la Escala de Coma de Glasgow inferior a 15) o si la presión arterial sistólica es inferior a 100 mmHg^(6)^. Se define como qSOFA positivo cuando se puntúan dos o más variables, y negativo cuando se puntúa una o ninguna^(6)^. La puntuación más alta (3 puntos) se asocia con un mal pronóstico^(13)^. Cuando la puntuación es ≥ 2, los estudios lo señalan como un indicador de potencial sepsis en pacientes con sospecha de infección fuera de la UCI^(13)^.

### Procesamiento y análisis de los datos

Los datos de la investigación fueron almacenados en la plataforma de recolección *REDCap*®^(11)^ y para procesar el análisis se utilizó el *Statistical Package for the Social Sciences* (SPSS), versión 23. El análisis descriptivo se realizó calculando la media, desviación estándar, mediana, mínimo y máximo. Para las variables categóricas se calcularon la frecuencia y el porcentaje. La asociación del qSOFA (positivo o negativo) con la aparición de deterioro clínico, comorbilidad, por categoría de clasificación de riesgo, desenlace dentro de las 24 horas posteriores al ingreso y desenlace final fue verificada mediante la prueba de chi-cuadrado y, cuando fue necesario, se utilizó la prueba exacta de Fisher. Se utilizó la prueba de Mann-Whitney o la prueba de Kruskal-Wallis para 3 categorías o más. Se calculó la sensibilidad y la especificidad del qSOFA para la aparición de deterioro clínico. Para todos los análisis, se consideró un valor de p< 0,05 para significación estadística. Se realizó regresión logística simple para verificar la relación de las variables independientes con relación al deterioro clínico (variable dependiente). Se realizó regresión logística múltiple para verificar el factor que mejor explicaba el “deterioro clínico”. El método de selección utilizado fue el *Forward*. El rendimiento del qSOFA para identificar el deterioro clínico al ingreso y antes del evento se analizó mediante el área bajo la curva ROC.

### Aspectos éticos

Esta investigación cumple con la Resolución 466/2012 del Consejo Nacional de Salud y fue aprobada por el Comité de Ética en Investigación de la institución, dictamen 4.919.847, Certificado de Presentación para la Apreciación Ética (CAAE, en portugués): 32684620.3.3001.0071, y por el Sistema Gestor de Proyectos de Investigación de la institución coparticipante, bajo dictamen n° 4316-20.

## Resultados

En esta investigación no hubo pérdidas durante el seguimiento. Se incluyeron 813 pacientes, con una edad promedio de 69 años (±17,8), y con predominio de hombres (61,5%, n= 500), blancos (97,2%, n= 769), católicos (73,7%, n= 583), casados (89,6%, n= 578) y asalariados (66%, n= 456). La mayoría de la población de estudio (75,3%, n= 612) buscó atención médica entre el primero y el sexto día del inicio de los síntomas, el 21,6% (n= 176) entre el séptimo y el décimo, y el 3,1% (n= 25) con más de 10 días de síntomas.

Con relación a la clasificación de riesgo, del total de 813 (n= 100%) pacientes, el 58,8% (n= 478) fueron clasificados como ESI 3, el 21,9% (n= 178) ESI 2, el 18% (n= 146) ESI 4 y el 1,4% (n= 11) ESI 1.

En cuanto a la aparición de deterioro clínico, el 16,7% (n= 136) de los individuos presentó insuficiencia respiratoria aguda dentro de las primeras 24 horas de ingreso al DE, el 0,7% (n= 6) shock, el 2,7% (n= 22) PCR y el 2,8% (n= 23) alteración del nivel de conciencia. Hubo 187 deterioros clínicos en las primeras 24 horas de ingreso al DE, según los datos de la Tabla 1.

**Tabla 1 -:** Deterioro clínico de pacientes con COVID-19 en las primeras 24 horas de ingreso al Departamento de Emergencias (n* = 813). São Paulo, SP, Brasil, 2022

**Deterioro clínico**	**24 horas desde el ingreso**
	n* (% ^†^ **)**
**Insuficiencia respiratoria aguda**	
No	677 (83,3)
Sí	136 (16,7)
**Shock**	
No	807 (99,3)
Sí	6 (0,7)
**Paro cardiorrespiratorio**	
No	791 (97,3)
Sí	22 (2,7)
**Alteración del nivel de conciencia**	
No	790 (97,2)
Sí	23 (2,8)

*n = Número absoluto; †% = Porcentaje

De la población estudiada (n= 813), el 69,7% (n= 567) presentaba alguna comorbilidad, siendo el 41% (n= 332) cardiovasculares, el 34,4% (n= 280) respiratorias, el 20,4% (n= 166) endocrinas, y el 10,8% (n= 88) neurológicas.

Los individuos con comorbilidades tuvieron mayor porcentaje de insuficiencia respiratoria, PCR y alteración del nivel de conciencia que aquellos sin comorbilidades (p< 0,0001). De los individuos que presentaron algún tipo de comorbilidad, 106 (n= 18,7%) presentaron insuficiencia respiratoria, 6 (n= 1,1%) shock, 22 (n= 3,9%) paro cardiorrespiratorio, y 20 (n= 3,5%) alteración del nivel de conciencia. Se observó que los pacientes con alguna comorbilidad tuvieron mayor porcentaje de deterioro clínico que aquellos sin (p< 0,0001), insuficiencia respiratoria aguda (p= 0,0019), shock (p= 0,0036), PCR (p= 0,0083) y cambio en el nivel de conciencia (p= 0,0007), al compararlos con aquellos que no tenían (p< 0,0001). Los pacientes con comorbilidades tuvieron mayor porcentaje de hospitalización en cuidados semiintensivos (p< 0,0001) y UCI (p< 0,0001), y mayor incidencia de mortalidad (p<0,0001). Las personas sin comorbilidades recibieron más altas hospitalarias en comparación con aquellas con comorbilidades (p< 0,0001) (Tabla 2).

**Tabla 2 -:** Asociación de la presencia de comorbilidades con deterioro clínico y desenlaces en la población estudiada (n* = 813). São Paulo, SP, Brasil, 2022

**Variables**	**Presencia de comorbilidades**		**p** ^†^ **-valor**
	No n* (% ^‡^ **)**	**Sí** n* (% ^‡^ **)**	
**Deterioro clínico al ingreso**			
No	213 (86,6)	413 (72,8)	<0,0001
Sí	33 (13,4)	154 (27,2)	
**Insuficiencia respiratoria**			
No	214 (87)	440 (77,6)	0,0019
Sí	32 (13)	127 (22,4)	
**Shock**			
No	244 (99,2)	539 (95,1)	0,0036
Sí	2 (0,8)	28 (4,9)	
**Paro cardiorrespiratorio**			
No	235 (95,5)	510 (89,9)	0,0083
Sí	11 (4,5)	57 (10,1)	
**Alteración del nivel de conciencia**			
No	239 (97,2)	513 (90,5)	0,0007
Sí	7 (2,8)	54 (9,5)	
**Desenlace en las primeras 24 horas**			
Alta	204 (82,9)	349 (61,6)	<0,0001
Ingreso Apartamento	20 (8,1)	35 (6,2)	
Ingreso Semiintensiva	12 (4,9)	86 (15,2)	
Ingreso UCI ^§^	10 (4,1)	77 (13,6)	
Óbito	0 (-)	20 (3,5)	
**Desenlace Final**			
Alta	235 (95,5)	469 (82,7)	<0,0001
Óbito	11 (4,5)	98 (17,3)	

*n = Número absoluto; †p = Nivel de significancia; ‡% = Porcentaje; §UCI = Unidad de Cuidados Intensivos

En cuanto a los resultados de los pacientes en las primeras 24 horas de ingreso al DE, 553 (n= 68%) fueron dados de alta, 98 (n= 12,1%) ingresaron en cuidados semiintensivos, 87 (n= 10,7%) ingresaron en la UCI, 55 (n= 6,8%) ingresaron en un apartamento, y 20 (n= 2,5) fallecieron.

Al asociarlo al primer deterioro clínico presentado en las primeras 24 horas, se encontró que los pacientes con qSOFA positivo al ingreso tuvieron mayor porcentaje de insuficiencia respiratoria y PCR que los pacientes con puntaje negativo con un nivel de significancia (p) < 0,0001 (Tabla 3).

**Tabla 3 -:** Asociación entre el *quick Sequential Organ Failure Assessment* (qSOFA*) y el deterioro clínico al ingreso e inmediatamente antes del evento (n† = 187). São Paulo, SP, Brasil, 2022

qSOFA*	**IRP** ^‡^	**Shock**	**PCR** ^§^	**ANC** ^║^		
	**n** ^†^ **(%** ^¶^ **)**	**n** ^†^ **(%** ^¶^ **)**	**n** ^†^ **(%** ^¶^ **)**	**n** ^†^ **(%** ^¶^ **)**	**Total**	**p** ** **valor**
**Al ingreso**						
Negativo	89 (11,9)	5 (0,7)	5 (0,7)	21 (2,8)	120	<0,0001
Positivo	47 (70,1)	1 (1,5)	17 (25,4)	2 (3)	67	
**Antes del deterioro**						
Negativo	83 (11,5)	5 (0,7)	5 (0,7)	16 (2,2)	109	<0,0001
Positivo	53 (59,6)	1 (1,1)	17 (19,1)	7 (7,9)	78	

*qSOFA = *quick Sequential Organ Failure Assessment*; †n = Número absoluto; ‡IRP = Insuficiencia respiratoria; §PCR = Paro cardiorrespiratorio; ║ANC = Alteración del nível de conciencia; ¶% = Porcentaje; **p = Nivel de significancia

Los pacientes con qSOFA negativo al ingreso tuvieron mayor porcentaje de ausencia de deterioro clínico que los pacientes con qSOFA positivo (p< 0,0001).

En cuanto a la categoría de clasificación de riesgo al ingreso, los pacientes que tuvieron qSOFA positivo presentaron mayor porcentaje de ESI 2 que aquellos con qSOFA negativo (p< 0,0001), y los pacientes con qSOFA negativo tuvieron mayor porcentaje de ESI 3 y ESI 4 (p< 0,0001).

Además, los pacientes con qSOFA negativo tuvieron un mayor porcentaje de alta que los pacientes con qSOFA positivo (p< 0,0001).

El qSOFA también mostró mayor especificidad y baja sensibilidad para rastrear el deterioro clínico, insuficiencia respiratoria, shock, PCR y alteración del nivel de conciencia (Tabla 4).

En regresión logística simple, la variable que mejor explicó el deterioro clínico fue el qSOFA. Los participantes con un qSOFA positivo al ingreso tenían 350 veces más probabilidades de experimentar deterioro en comparación con los participantes con un qSOFA negativo. Los participantes con un qSOFA positivo antes del evento tenían 40 veces más probabilidades de experimentar deterioro en comparación con los participantes con un qSOFA negativo. Por otro lado, en regresión logística múltiple, la variable que mejor explicó el deterioro fue el qSOFA al ingreso: los pacientes con un qSOFA positivo tenían 352 veces más probabilidades de empeorar en comparación con los pacientes con un qSOFA negativo (p <0,0001 IC de 95% [48,46; 2562,12]).

Al analizar el desempeño del qSOFA para identificar el deterioro, el área bajo la curva ROC fue de 0,68 (0,63 – 0,73) en el momento del ingreso del paciente, y de 0,70 (0,65 – 0,75) inmediatamente antes del evento de ingreso, como se muestra en la Figura 1.

**Tabla 4 -:** Sensibilidad y especificidad del *quick Sequential Organ Failure Assessment* (qSOFA*) para identificar el deterioro clínico antes del evento (n† = 813). São Paulo, SP, Brasil, 2022

**Tipos de deterioro clínico**	**Deterioro clínico**			
	**No**	**Sí**	**Sensibilidad**	**Especificidad**
	**n** ^†^ **(%** ^‡^ **)**	**n** ^†^ **(%** ^‡^ **)**	**(%** ^‡^ **)**	**(%** ^‡^ **)**
**Deterioro clínico en el Departamento de Emergencias**				
qSOFA * Negativo	615 (75,6)	109 (13,4)	41,7	98,2
qSOFA * Positivo	11 (1,4)	78 (9,6)		
**Insuficiencia respiratoria**				
qSOFA * Negativo	627 (77,1)	97 (11,9)	39,0	95,9
qSOFA * Positivo	27 (3,3)	62 (7,6)		
**Shock**				
qSOFA * Negativo	696 (85,6)	28 (3,4)	6,7	88,9
qSOFA * Positivo	87 (10,7)	2 (0,2)		
**Paro cardiorrespiratorio**				
qSOFA * Negativo	679 (83,5)	45 (5,5)	33,8	91,3
qSOFA * Positivo	66 (8,1)	23 (2,8)		
**Alteración del nivel de conciencia**				
qSOFA * Negativo	683 (84)	41 (5,0)	32,8	90,8
qSOFA * Positivo	69 (8,5)	20 (2,5)		

*qSOFA = *quick Sequential Organ Failure Assessment*; †n = Número absoluto; ‡% = Porcentaje

**Figura 1 -:** Curva ROC de qSOFA al ingreso e inmediatamente antes del deterioro clínico

Según el análisis de la curva ROC, el punto de corte de deterioro clínico fue ≥ 1, tanto al ingreso como inmediatamente antes del deterioro clínico. Al ingreso, el qSOFA tenía una sensibilidad del 66% y una especificidad del 55%, e inmediatamente antes del deterioro clínico, una sensibilidad del 48% y una especificidad del 88%.

## Discusión

En este estudio, evaluamos el desempeño de qSOFA como herramienta predictiva del deterioro clínico en pacientes con COVID-19 que fueron tratados en un DE. El uso de puntuaciones de alerta temprana ha ido ganando protagonismo en el triaje de pacientes con riesgo de deterioro clínico^(14)^, y su uso parece razonable, ya que la OMS ha emitido advertencias sobre los riesgos de futuras pandemias^(15)^. El qSOFA fue desarrollado y recomendado como un sistema de evaluación clínica para estratificar a los pacientes según la gravedad de la sepsis^(16)^. Una puntuación qSOFA de 2 o más puede indicar un pronóstico desfavorable^(17)^.

La edad promedio en este estudio fue mayor que el promedio mundial^(18)^. La mayoría de nuestros pacientes fueron clasificados en las categorías ESI 3 y ESI 2, que indican que el paciente debe ser atendido en 15 minutos, ya que se considera muy urgente o necesita utilizar algún recurso^(4)^. Pocos estudios han asociado los sistemas de detección con desenlaces o han sido validados para este escenario. Un estudio retrospectivo, que utilizó el Sistema de Triaje Manchester y fue desarrollado en el sur de Brasil con 1228 pacientes, tuvo hallazgos similares, es decir, la mayoría de los pacientes con COVID-19 fueron clasificados como muy urgentes y urgentes^(19)^. Este hecho puede estar asociado a no buscar atención médica ante la presencia de síntomas leves, según las directrices sanitarias de la época^(20)^.

El *Centers for Disease Control and Prevention* emitió un informe de los primeros meses de la pandemia sobre 1,3 millones de casos notificados, lo que indica una tasa de mortalidad de alrededor del 2,5%, siendo la mayoría de los infectados oligosintomáticos o asintomáticos. Sin embargo, aproximadamente el 14% de la población experimentó síntomas más agudos de neumonía viral y el 2% pudo desarrollar una forma grave de la enfermedad que requirió cuidados intensivos^(21)^. La mayoría de los pacientes que acudieron a urgencias fueron dados de alta, pero observamos una relación entre la edad y las comorbilidades con desenlaces negativos. Sin embargo, más del doble fueron hospitalizados y, entre ellos, el 41% ingresó en la UCI o falleció dentro de las primeras 24 horas. Estas diferencias se pueden explicar, en parte, porque la edad promedio de nuestra población era superior a la media global, además de que casi el 70% presentaba al menos una comorbilidad, especialmente cardiovascular. La relación entre grupo de edad, presencia de comorbilidades y gravedad de la enfermedad ya está bien establecida. Un estudio de caracterización demostró que los octogenarios tenían 20 veces más probabilidades de morir en comparación con el grupo de edad de 50 a 59 años^(22)^. Otro estudio demostró 6 veces más hospitalizaciones y 12 veces más muertes entre aquellos con comorbilidades^(21)^.

El período de mayor gravedad de la enfermedad generalmente comienza después del 6º día del inicio de los síntomas, ya que se espera haber alcanzado el pico de la carga viral en el huésped^(23-24)^, y es cuando la mayoría de los pacientes se presentan en el DE con signos de neumonía viral. La mayoría de nuestros pacientes buscaron atención dentro de los primeros 6 días de presentar síntomas, lo que nos llama la atención sobre la posibilidad de un deterioro clínico en los próximos días de su hospitalización, ya que el desarrollo del Síndrome de Dificultad Respiratoria Aguda ocurre entre los días 7 y 9^(24)^. De hecho, entre los 260 pacientes hospitalizados, 187 pacientes presentaron algún tipo de deterioro clínico en las primeras 24 horas, siendo la insuficiencia respiratoria la condición más prevalente, presentándose en el 72,7% de los casos de deterioro clínico. El SARS-CoV2, al llegar a las vías respiratorias inferiores, infecta especialmente a los neumocitos T2 (células que expresan la enzima convertidora de angiotensina 2 en grandes cantidades), provocando neumonía viral y, en algunos casos, shock secundario al Síndrome de Respuesta Inflamatoria Sistémica (SRIS)^(24)^. Varios informes señalan la insuficiencia respiratoria como el deterioro clínico más prevalente en pacientes con COVID-19^(2,24-25)^.

En un escenario de urgencias, especialmente en periodos de pico y sobrecarga, tanto para los pacientes que reciben atención como para los ingresados en espera de una cama, pueden ocurrir situaciones de monitorización insuficiente. En este contexto, es oportuno implementar sistemas de seguimiento y respuesta a emergencias^(26)^. Se adaptaron varios sistemas de detección para tener en cuenta la característica típica de la enfermedad: signos de insuficiencia respiratoria^(3,27-28)^.

El qSOFA es una de esas herramientas, validado en el campo para rastrear los signos clínicos de sepsis. Al tratarse de una puntuación sencilla aplicada a pie de cama, que en la práctica facilita la adherencia, nos parece adecuada su validación en diferentes escenarios, como en el seguimiento de la insuficiencia respiratoria por neumonía viral^(17)^.

En esta casuística, el qSOFA positivo se asoció con ESI 2, una mayor tasa de deterioro clínico, ingreso en UCI y mortalidad. El qSOFA mostró alta especificidad, pero baja sensibilidad para todos los tipos de deterioro clínico. Los pacientes con un qSOFA positivo al ingreso tenían 350 veces más probabilidades de experimentar algún deterioro clínico en comparación con aquellos con un qSOFA negativo. En la curva ROC, el punto de corte de deterioro clínico fue ≥ 1, tanto al ingreso como inmediatamente antes del evento, encontrándose mejor sensibilidad (66%) al ingreso, y especificidad (88%) inmediatamente antes del evento.

Nuestros hallazgos, cuando se contextualizan, tienen sentido, ya que el qSOFA tiene el recuento de la frecuencia respiratoria como el único patrón temprano de insuficiencia respiratoria, el deterioro clínico más prevalente en nuestra población y en la literatura^(3,27-28)^.

Con relación al desempeño, una revisión sistemática analizó el desempeño de varios puntajes con la mortalidad, y el qSOFA obtuvo un puntaje de 0.622 en la curva ROC, mostrando un desempeño menor; sin embargo, no lo analizó en términos de deterioro clínico^(29)^. Llamamos la atención sobre el verdadero propósito de las puntuaciones de alerta temprana que, cuando se diseñan y desarrollan, tienen la función de rastrear el empeoramiento clínico, y no de predecir la mortalidad^(14)^. En nuestro estudio, el qSOFA fue de 0,68 (95%;0,63-0,73) al ingreso, e inmediatamente antes del evento fue mayor, 0,70 (95%;0,65-0,75), lo que puede considerarse una herramienta útil, especialmente a la hora de reducir el punto de corte.

Al parecer, el qSOFA sirvió como un puntaje práctico con buenos resultados para uso de emergencia en pacientes con COVID-19, siendo una prueba clínicamente útil, según resultados del área bajo la curva ROC.

Este estudio es importante para la práctica clínica, ya que tiene como objetivo aumentar la seguridad en salud mediante la identificación de una puntuación de deterioro clínico para su uso en DE, que presenta un escenario de sobrepoblación a nivel mundial. En este estudio, el qSOFA tuvo una alta especificidad, pero una baja sensibilidad en pacientes con COVID-19, y no debe usarse solo.

Para maximizar la seguridad, se destaca la importancia de sistematizar la evaluación, por medio de puntajes de alerta temprana. Son fáciles de aplicar, con datos que se miden de forma rutinaria en todos los pacientes. Los hallazgos de este estudio pueden ayudar a los profesionales a crear estrategias para organizar el flujo de atención, con el fin de mejorar el proceso, no solamente en el DE, sino también en las unidades de internación.

Este estudio tuvo la limitación de retratar la realidad de un único centro de una institución filantrópica, con población principalmente de medicina privada, lo que puede restringir la generalización de los resultados.

## Conclusión

El qSOFA mostró una especificidad superior al 98%; sin embargo, demostró una baja sensibilidad para rastrear el deterioro clínico. Esto indica que no debe utilizarse de forma aislada en la práctica asistencial, pero tiene potencial para formar parte de la evaluación clínica de pacientes adultos con COVID-19 en el Departamento de Emergencias.
